# Prognostic utility of Wnt/β-catenin signaling pathway biomarkers in predicting outcomes of patients with ST-segment elevation myocardial infarction

**DOI:** 10.3389/fcvm.2026.1746054

**Published:** 2026-03-02

**Authors:** Lei Wang, Chengmin Tao, Lingfei Yang

**Affiliations:** 1 Department of Cardiovascular Medicine, The Second Affiliated Hospital of Wannan Medical College, Wuhu, Anhui, China; 2 Department of Cardiovascular Medicine, Wuhu First People’s Hospital, Wuhu, Anhui, China

**Keywords:** biomarker, major adverse cardiovascular events, prognosis, ST-segment elevation myocardial infarction, Wnt/β-catenin signaling

## Abstract

**Background:**

To investigate the expression profiles of Wnt/β-catenin signaling pathway–related biomarkers in patients with ST-segment elevation myocardial infarction (STEMI) and to evaluate their predictive value for major adverse cardiovascular events (MACE).

**Methods:**

A total of 200 STEMI patients admitted to the emergency department between August 2021 and August 2024 were enrolled. Peripheral venous blood samples were collected immediately before emergency percutaneous coronary intervention (PCI) and serum levels of Wnt1, β-catenin, Wnt5a, secreted frizzled-related protein 5 (SFRP5), and Dickkopf-related protein 1 (DKK1) were measured using enzyme-linked immunosorbent assay. Based on the occurrence of MACE during hospitalization and within 1-year follow-up, patients were categorized into the MACE group (*n* = 29) and non-MACE group (*n* = 171). Kaplan–Meier survival analysis was used to assess differences in prognosis among patients with different biomarker levels. Univariate and multivariate Cox proportional hazards regression analyses were performed to identify independent risk factors for MACE.

**Results:**

Serum levels of Wnt1, β-catenin, Wnt5a, and DKK1 were elevated, whereas SFRP5 was markedly reduced in the MACE group. Patients with high serum levels of Wnt1, β-catenin, Wnt5a, or DKK1 had significantly higher MACE incidence rates, while those with high SFRP5 levels had a lower incidence. β-catenin, DKK1, and cardiac troponin I (cTnI) were identified as independent risk factors for MACE in STEMI patients. The combined prediction model of β-catenin and DKK1 (AUC = 0.946; 95% CI = 0.910–0.981) showed superior predictive performance compared with single biomarkers.

**Conclusion:**

The combined detection of β-catenin and DKK1 may serve as a potential biomarker for risk stratification in STEMI patients.

## 1 Introduction

Acute coronary syndrome (ACS) remains the leading cause of cardiovascular mortality worldwide. Among its subtypes, ST-segment elevation myocardial infarction (STEMI) represents the most critical and life-threatening form. The underlying pathology of STEMI involves rupture of an atherosclerotic coronary plaque, triggering acute thrombosis that abruptly interrupts myocardial blood flow and causes ischemic necrosis of cardiomyocytes (1). Although emergency percutaneous coronary intervention (PCI) effectively restores myocardial perfusion by reopening the infarct-related artery and has markedly reduced acute-phase mortality, the risk of adverse cardiovascular outcomes, such as cardiac death, recurrent myocardial infarction, malignant arrhythmias, and heart failure rehospitalization, remains substantial in the post-procedural period (2). Therefore, in the early emergency stage, identifying reliable biomarkers that can accurately and promptly stratify high-risk patients is of great clinical importance for implementing individualized interventions and improving prognosis.

Currently, clinical assessment of STEMI prognosis primarily relies on risk scoring systems and traditional biomarkers. Risk models such as the Global Registry of Acute Coronary Events (GRACE) score and Thrombolysis in Myocardial Infarction (TIMI) score integrate clinical indicators including age, blood pressure, heart rate, and renal function for risk stratification, but they lack sufficient dynamic sensitivity (3, 4). The conventional biomarker cardiac troponin I (cTnI) serves as a sensitive diagnostic indicator for myocardial infarction; however, its specificity for prognostic prediction is limited (5). Elevated peak cTnI levels can only partially explain the prognostic heterogeneity among STEMI patients and may yield false-positive results under conditions such as renal insufficiency or infection, limiting its value as a stand-alone prognostic marker (6, 7). Consequently, the discovery of novel biomarkers with both high specificity and early detectability has become a key focus in contemporary STEMI research.

In recent years, the roles of molecular signaling pathways in the onset and progression of myocardial infarction have attracted increasing attention. The Wnt/β-catenin signaling pathway, a conserved pathway essential for embryonic cardiac development, plays crucial roles in embryogenesis, tissue repair, and disease pathogenesis (8–10). Previous studies have demonstrated that the Wnt/β-catenin pathway remains largely quiescent in the adult heart (11, 12). However, following acute myocardial ischemic injury, this pathway becomes reactivated and participates extensively in regulating inflammation, apoptosis, fibrosis, and ventricular remodeling (13–15). Key components of this pathway, including Wnt ligands (e.g., Wnt1 and Wnt5a) that initiate signal transduction (16, 17), the central effector β-catenin, and endogenous antagonists such as Dickkopf-related protein 1 (DKK1) and the secreted frizzled-related protein (SFRP) family (18–20), collectively maintain a dynamic balance that may influence the direction and quality of myocardial repair. Moreover, modulation of the Wnt/β-catenin signaling pathway has been shown to mitigate myocardial infarction and improve cardiac outcomes in experimental models (21–23). Nevertheless, comprehensive clinical evidence regarding the expression profiles of key Wnt/β-catenin pathway molecules in STEMI patients and their associations with clinical prognosis remains limited.

Therefore, this study aims to measure serum levels of Wnt1, β-catenin, Wnt5a, SFRP5, and DKK1 in STEMI patients, to delineate their expression characteristics between those with and without major adverse cardiovascular events (MACE). Furthermore, the study sought to evaluate the independent predictive value of these biomarkers for MACE, with the goal of identifying novel and forward-looking biological indicators for risk assessment and prognostic prediction in STEMI patients.

## 2 Materials and methods

### 2.1 Study population

This was a single-center, retrospective observational cohort study. We screened patients with STEMI admitted between August 2021 and August 2024. To ensure a complete 12-month follow-up for all included subjects, the follow-up data were locked and analyzed in August 2025. Patients were eligible if they met all the following criteria.

Inclusion criteria: (1) Meeting the diagnostic criteria of the European Society of Cardiology (ESC) Guidelines for the Management of Acute STEMI (24), specifically: (i) typical chest pain lasting ≥30 min not relieved by nitroglycerin; (ii) ST-segment elevation in at least two contiguous leads (≥0.20 mV in precordial leads or ≥0.10 mV in limb leads) on electrocardiogram; (iii) cardiac troponin I (cTnI) on admission > the 99th percentile upper reference limit; (2) Received emergency PCI within 12 h of symptom onset; (3) Had residual serum samples stored in the −80℃ biobank; (4) Had completed at least 12 months of follow-up by the time of data lock (August 2025).

Exclusion criteria: (1) Concomitant severe hepatic or renal dysfunction, malignant tumor, or autoimmune disease; (2) History of prior myocardial infarction, heart failure, or valvular heart disease; (3) Use of glucocorticoids or immunosuppressants within 3 months before admission; (4) Coagulation disorders or active bleeding.

**Figure 1:**
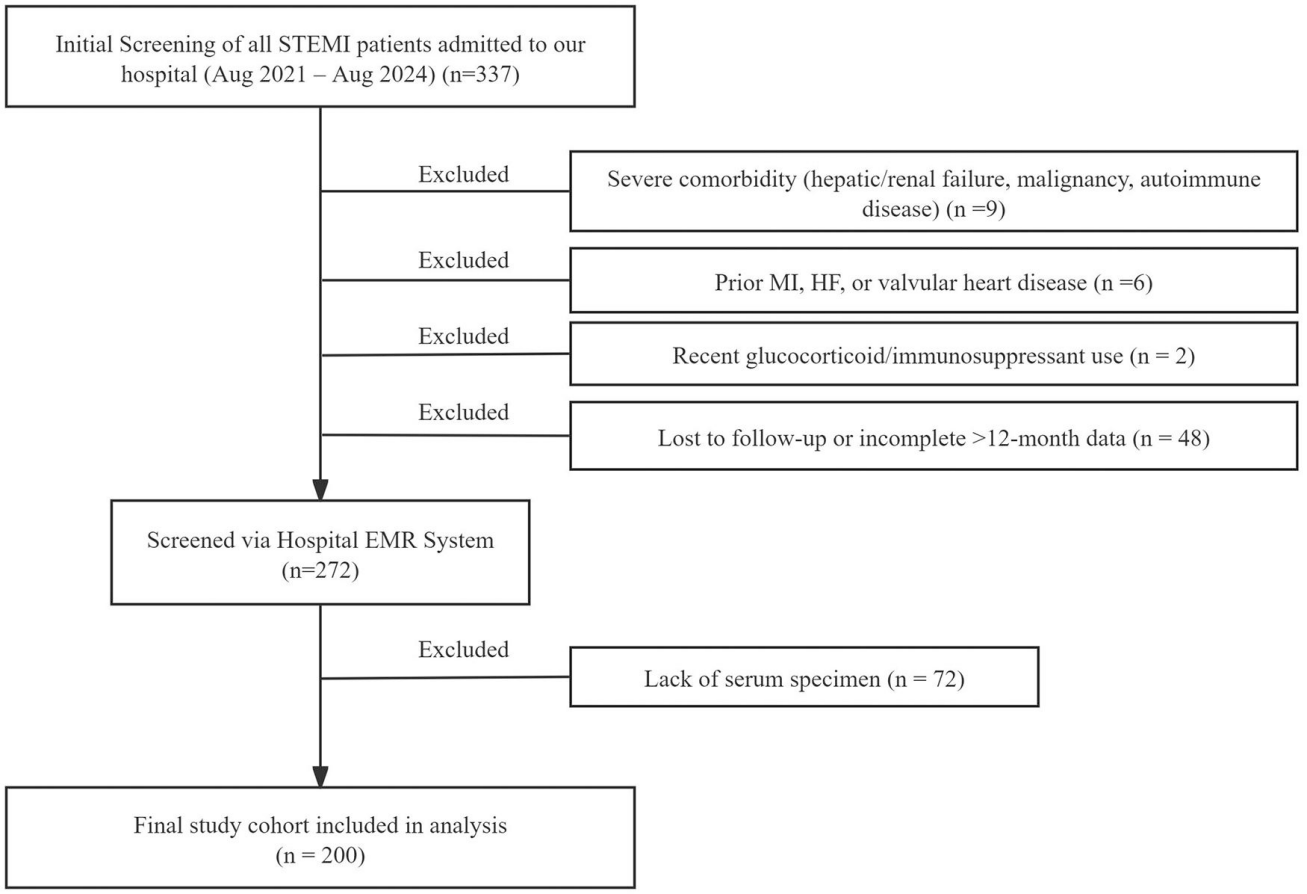
Patient screening flowchart.

According to the above criteria, a total of 200 consecutive patients were included in the final analysis (Figure 1). This study was approved by the Institutional Medical Ethics Committee of The Second Affiliated Hospital of Wannan Medical College. Due to its retrospective design, the requirement for individual written informed consent was waived by the Ethics Committee; all patient data were anonymized for analysis.

### 2.2 Baseline data collection

Baseline data were retrospectively extracted from the hospital electronic medical record system and included: age, sex, smoking history (defined as having smoked a total of ≥100 cigarettes in one's lifetime) (25), comorbidities (hypertension, diabetes mellitus, hyperlipidemia), systolic and diastolic blood pressure and heart rate at admission, cTnI, and high-sensitivity cardiac troponin (hs-cTn).

### 2.3 Biomarker measurement

Prior to emergency PCI and after hospital admission, 5 mL of venous blood was drawn from the antecubital vein by a trained nurse. Blood samples were allowed to stand, then centrifuged to separate serum, which was aliquoted and stored at −80 °C until assay.

Serum concentrations of Wnt1, β-catenin, Wnt5a, DKK1, and SFRP5 were measured by enzyme-linked immunosorbent assay (ELISA). All ELISA kits were purchased from Shanghai Enzyme-linked Biotechnology Co., Ltd. (Shanghai, China) and assays were performed strictly according to the manufacturers' instructions. The plate reader used was a Bio-Tek ELx800 microplate reader (Bio-Tek Instruments, Inc., USA). Each sample was assayed in triplicate and the mean of three measurements was recorded as the final value. To evaluate the precision of the ELISA measurements, both intra-assay and inter-assay variability were assessed. For intra-assay variability, the coefficient of variation (CV) was calculated from the triplicate measurements of each sample within the same assay plate. The coefficient of variation (CV) was calculated using the formula: CV (%) = (SD/Mean) × 100%. The mean intra-assay CVs for all biomarkers were below 7.83%. For inter-assay variability, a pooled serum sample was included as a control in each independent assay run. The CV calculated from these control measurements across different plates and days was below 11.27% for all analytes. These results confirm the acceptable reproducibility and precision of our ELISA measurements. Laboratory technicians performing the ELISA assays were blinded to all patient clinical information and outcomes.

### 2.4 Follow-up and endpoint definition

Endpoint events were ascertained through a structured review of the hospital's comprehensive electronic health records, readmission databases, outpatient clinic files, and telephone follow-up records. Patients were followed by telephone monthly and with outpatient visits at 3 months, 6 months, and 12 months post-procedure. Loss to follow-up was defined as inability to contact the patient for two consecutive scheduled contacts or patient refusal to continue follow-up.

The primary endpoint was occurrence of major adverse cardiovascular events (MACE) within 1 year, defined as cardiovascular death, non-fatal recurrent myocardial infarction, target-vessel revascularization, or rehospitalization for heart failure.

Cardiovascular death required death certificate documentation or confirmation by next of kin. Recurrent myocardial infarction and target-vessel revascularization required hospital records or coronary angiography reports. Heart failure hospitalization required echocardiography reports and inpatient records. All suspected endpoint events were submitted to an independent, blinded endpoint adjudica tion committee (two cardiologists and one radiology/imaging specialist). After review of de-identified medical documents, events were adjudicated by discussion and majority vote. Committee members were fully blinded to the patients' biomarker levels and the study hypothesis during the adjudication process.

### 2.5 Statistical analysis

Statistical analyses were performed using SPSS version 26.0 (IBM Corp., Armonk, NY, USA) and R version 4.2.1 (R Foundation for Statistical Computing, Vienna, Austria). Continuous variables were first tested for normality using the Shapiro–Wilk test. Normally distributed continuous variables are presented as mean ± standard deviation (SD) and compared between groups using independent-samples t-test. Non-normally distributed continuous variables are presented as median (interquartile range) and compared using the Mann–Whitney U test. Categorical variables are expressed as counts (percentages) and compared using the *χ*^2^ test. Survival analyses were conducted with time to first Major Adverse Cardiovascular Event (MACE) within 1 year as the primary endpoint. Survival curves were generated using the Kaplan–Meier method and compared by the log-rank test. Independent predictors of short-term prognosis in STEMI patients were identified by multivariable Cox proportional hazards regression analysis. The variables included in the initial Cox model were selected based on clinical relevance and univariate screening (*P* < 0.1). The proportional hazards assumption was verified using Schoenfeld residuals. Multicollinearity was assessed by variance inflation factors (VIF), and all retained variables had VIF < 2.0. Model results are presented as hazard ratios (HR) with 95% confidence intervals (CI). Receiver operating characteristic (ROC) curve analysis was used to assess the predictive performance of biomarkers for MACE; area under the curve (AUC) and 95% confidence intervals (95% CI) were calculated. The optimal cut-off point for the combined biomarker model was determined by maximizing the Youden index (sensitivity + specificity-1). A two-tailed *P* value < 0.05 was considered statistically significant.

## 3 Results

### 3.1 Baseline characteristics

Comparison of baseline characteristics showed that the MACE group had significantly higher levels of cTnI, hs-cTn, and heart rate than the non-MACE group (*P* < 0.05). Significant differences were also observed in age, smoking history, and diabetes (all *P* < 0.05). No significant differences were found in sex, hypertension, or hyperlipidemia between the two groups (*P* > 0.05) (Table 1).

**Table 1:** Comparison of baseline characteristics between the two groups.

Variables		MACE group	Non-MACE group	*t/*χ^2^	*P*
		(*n* = 29)	(*n* = 171)		
Age/(year)		63.69 ± 6.00	54.25 ± 6.41	7.4	<0.001
Sex (*n*/%)	Male	21 (72.41%)	121 (70.76%)	0.033	0.856
	female	8 (27.59)	50 (29.24%)		
Hypertension (*n*/%)	Yes	18 (62.07%)	90 (52.63%)	0.9	0.346
	No	11 (37.93%)	81 (47.09%)		
Hyperlipidemia (*n*/%)	Yes	16 (55.17%)	62 (36.26%)	3.73	0.053
	No	13 (44.83%)	158 (63.74%)		
Diabetes (*n*/%)	Yes	15 (51.72%)	45 (26.32%)	7.62	0.006
	No	14 (48.28%)	126 (73.68%)		
Smoking (*n*/%)	Yes	21 (72.41%)	49 (28.65%)	20.87	<0.001
	No	8 (27.59%)	122 (71.35%)		
Heartrate (bpm)		94.78 ± 14.14	79.55 ± 11.31	6.45	<0.001
Systolic blood pressure (mmHg)		128.55 ± 19.78	135.67 ± 15.79	1.84	0.074
Diastolic blood pressure (mmHg)		77.93 ± 13.20	80.63 ± 10.07	1.05	0.302
cTnI (ng/L)		132.50 ± 8.29	122.17 ± 7.05	1.221	<0.001
hs-cTn (ng/L)		423.14 ± 195.81	273.00 ± 134.72	3.97	<0.001

### 3.2 Comparison of biomarker levels between prognostic groups

ELISA results revealed significant differences in the serum levels of Wnt1, β-catenin, Wnt5a, SFRP5, and DKK1 between the two groups (all *P* < 0.001). Compared with the non-MACE group, the MACE group exhibited significantly higher serum levels of Wnt1, β-catenin, Wnt5a, and DKK1, whereas SFRP5 levels were markedly lower (Table 2).

**Table 2:** Comparison of biomarker levels between prognostic groups.

Variables		MACE group	Non-MACE group	*P*
		(*n* = 29)	(*n* = 171)	
Wnt1 (ng/mL)	3.09 ± 0.51	2.29 ± 0.37	8.08	<0.001
β-catenin (ng/mL)	4.02 ± 0.52	3.33 ± 0.49	6.96	<0.001
Wnt5a (ng/mL)	0.63 ± 0.18	0.37 ± 0.13	7.64	<0.001
SFRP5 (ng/mL)	37.58 ± 6.3	44.82 ± 5.6	6.31	<0.001
DKK1 (ng/mL)	14.36 ± 0.66	12.95 ± 0.89	7.76	<0.001

### 3.3 Association between Wnt/β-catenin pathway biomarkers and prognosis

**Figure 2:**
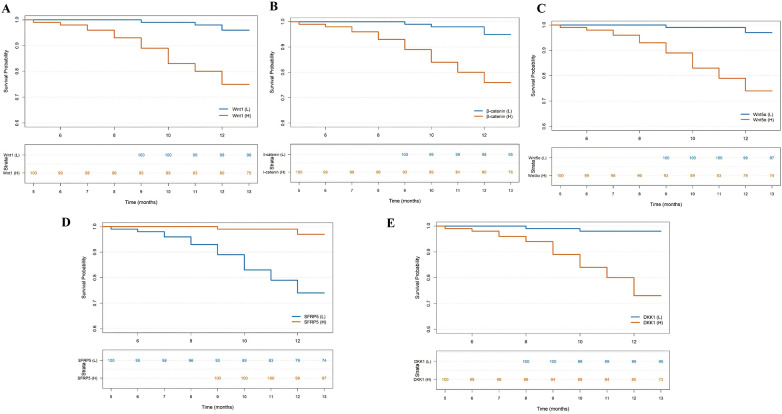
Kaplan–Meier survival analysis. **(A)** Kaplan–Meier analysis for different Wnt1 expression levels. **(B)** Kaplan–Meier analysis for different β-catenin expression levels. **(C)** Kaplan–Meier analysis for different Wnt5a expression levels. **(D)** Kaplan–Meier analysis for different SFRP5 expression levels. **(E)** Kaplan–Meier analysis for different DKK1 expression levels.

During the 12-month follow-up, 29 of 200 patients (14.5%) experienced MACE. Based on the median expression levels of Wnt1 (2.42 ng/mL), β-catenin (3.41 ng/mL), Wnt5a (0.40 ng/mL), SFRP5 (43.53 ng/mL), and DKK1 (13.21 ng/mL), patients were divided into high- and low-expression groups. Kaplan–Meier analysis showed that the incidence of MACE was significantly higher in the high-expression groups of β-catenin, Wnt1, Wnt5a, and DKK1, and significantly lower in the high-expression group of SFRP5 (Table 3; Figure 2).

**Table 3:** Incidence of MACE according to different biomarker expression levels (*n*, %).

Variables		MACE group	Non-MACE group	Log rank (Mantel-Cox)	*P*
		(*n* = 29)	(*n* = 171)		
Wnt1	Wnt1 (L)	4 (13.79%)	96 (56.14%)	18.144	<0.001
	Wnt1 (H)	25 (86.21%)	75 (43.85%)		
β-catenin	β-catenin (L)	5 (17.24%^)	95 (55.56%)	15.012	<0.001
	β-catenin (H)	24 (82.75%)	76 (44.44%)		
Wnt5a	Wnt5a (L)	3 (10.34%)	97 (45.03%)	21.64	<0.001
	Wnt5a (H)	26 (89.66%)	74 (43.27%)		
SFRP5	SFRP5 (L)	26 (89.66%)	74 (43.27%)	21.64	<0.001
	SFRP5 (H)	3 (10.34%)	97 (45.03%)		
DKK1	DKK1 (L)	2 (6.89%)	98 (57.31%)	24.93	<0.001
	DKK1 (H)	27 (93.10%)	73(42.69%)		

### 3.4 Cox regression analysis of independent predictors for MACE

To evaluate the independent predictive value of Wnt/β-catenin pathway biomarkers, variables with *P* < 0.1in univariate analysis (age, diabetes, hyperlipidemia, smoking history, heart rate, cTnI, hs-cTn, Systolic blood pressure, Wnt1, β-catenin, Wnt5a, SFRP5, and DKK1) were included in a multivariate Cox regression model. After adjustment, β-catenin, DKK1, and cTnI were identified as independent risk factors for MACE within 12 months in STEMI patients (*P* < 0.01) (Table 4).

**Table 4:** Cox regression analysis of clinicopathological factors associated with MACE in STEMI patients.

Variables	*β*	SE	Wald	Exp (*β*)	95% CI	*P*
Age	0.051	0.032	2.576	1.053	0.989–1.121	0.108
Diabetes	−0.748	0.526	2.020	0.473	0.169–1.328	0.155
Hyperlipidemia	−0.248	0.526	0.223	0.780	0.278–2.187	0.637
Smoking	−0.546	0.637	0.735	0.579	0.166–2.019	0.391
Heartrate	−0.004	0.020	0.031	0.996	0.957–1.037	0.86
Systolic blood pressure	0.013	0.017	0.609	1.013	0.981–1.046	0.435
hs-cTn	0.001	0.001	0.935	1.001	0.999–1.004	0.334
cTnI	0.099	0.032	9.752	1.104	1.038–1.175	0.002
Wnt1	0.345	0.527	0.427	1.412	0.502–3.969	0.513
β-catenin	2.423	0.747	10.515	11.274	2.607–48.754	0.001
Wnt5a	2.364	1.679	1.982	10.633	0.396–25.647	0.159
SFRP5	−0.045	0.041	1.245	0.956	0.883–1.035	0.265
DKK1	0.973	0.411	5.588	2.645	1.181–5.925	0.018

### 3.5 Predictive performance of biomarkers for MACE

**Figure 3:**
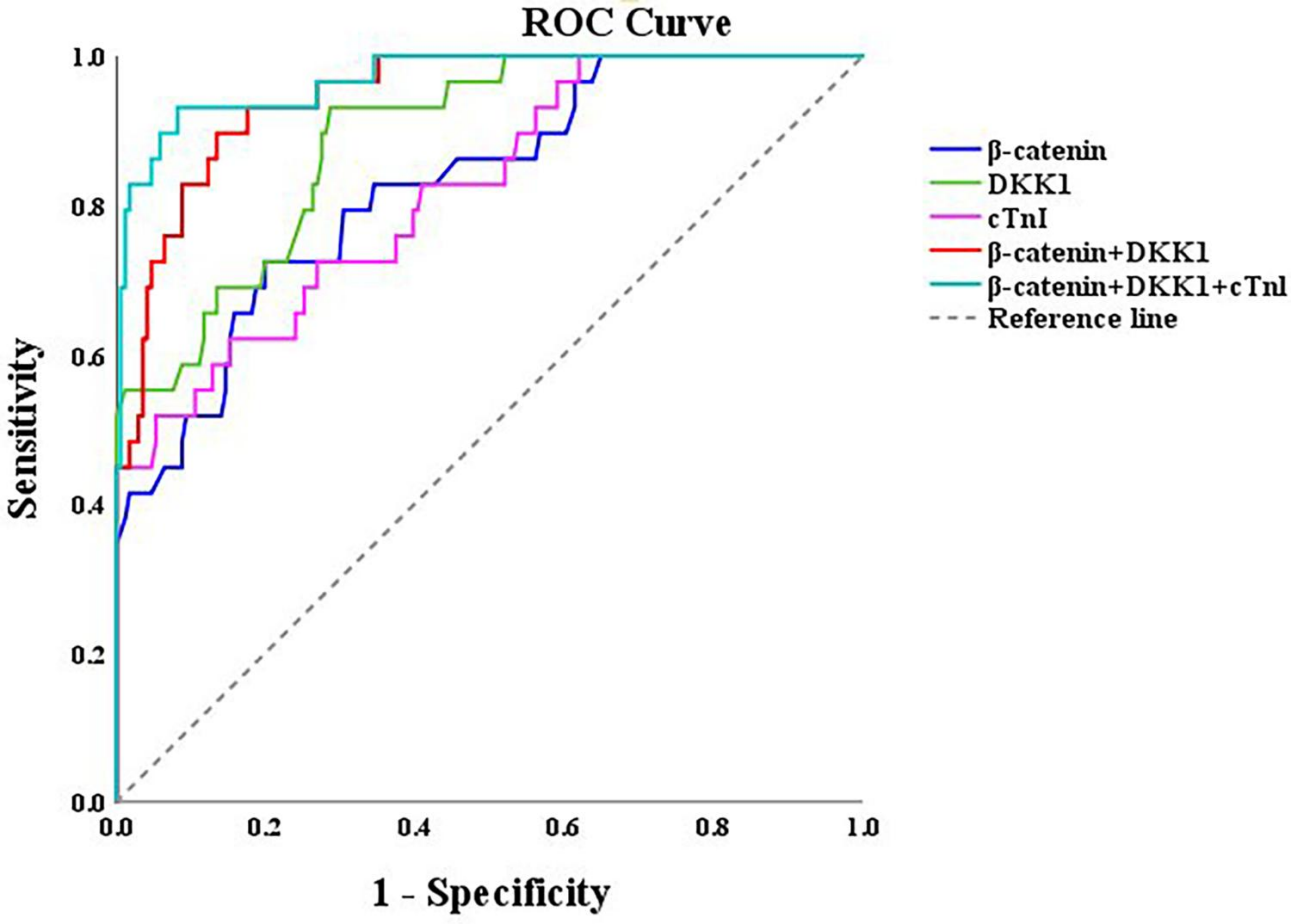
ROC curves of clinicopathological factors predicting MACE in STEMI patients.

ROC curve analysis demonstrated that β-catenin, DKK1, and cTnI predicted 12-month MACE in STEMI patients with AUCs of 0.826 (95% CI: 0.744–0.807, *P* < 0.001), 0.890 (95% CI: 0.832–0.949, *P* < 0.001), and 0.819 (95% CI: 0.735–0.903, *P* < 0.001), respectively. The optimal cut-off values for β-catenin and DKK1 were 3.835 ng/mL and 13.575 ng/mL, respectively. Combined detection of β-catenin and DKK1 further improved the AUC to 0.946 (95% CI: 0.910–0.981, *P* < 0.001), indicating superior predictive performance compared with single biomarkers (Table 5; Figure 3).

**Table 5:** ROC curve analysis of clinicopathological factors associated with MACE in STEMI patients.

Variables	AUC (95%CI)	Accuracy	Sensitivity	Specificity	PPV	NPV	*P*
β-catenin	0.826 (0.744–0.807)	0.790	0.724	0.801	0.382	0.945	<0.001
DKK1	0.890 (0.832–0.949)	0.745	0.931	0.713	0.355	0.984	<0.001
cTnl	0.819 (0.735–0.903)	0.815	0.621	0.848	0.409	0.929	<0.001
β-catenin + DKK1	0.946 (0.910–0.981)	0.870	0.897	0.865	0.531	0.980	<0.001
β-catenin + DKK1 + cTnl	0.969 (0.939–0.999)	0.920	0.931	0.918	0.659	0.987	<0.001

## 4 Discussion

Myocardial repair following STEMI is a highly complex biological process involving intricate regulation by multiple molecular pathways. Among these, the Wnt/β-catenin signaling pathway, an essential regulator of cardiac development during embryogenesis, has recently attracted attention for its role in myocardial injury and repair in the adult heart (12). While prior studies have established associations between individual Wnt pathway components and cardiovascular diseases, the fundamentally novel aspect of this study lies in the systematic, parallel evaluation of a panel of key Wnt pathway biomarkers (including both activators and antagonists) in the acute phase of STEMI, and in the development of a combined predictive model based on their interaction. Our aim was to elucidate the association between these pathway-related biomarkers and the occurrence of MACE, thereby identifying novel prognostic targets and enriching current evidence on the role of the Wnt/β-catenin pathway in acute ischemic heart disease.

We found that serum levels of Wnt1, β-catenin, and Wnt5a were significantly elevated in the MACE group, consistent with previous findings on the involvement of the Wnt/β-catenin pathway in cardiovascular diseases (18). This pathway plays a crucial role in regulating cell proliferation, differentiation, and inflammation. Following acute MI, ischemia, hypoxia, and subsequent inflammation disrupt myocardial homeostasis, leading to reactivation of the otherwise quiescent Wnt/β-catenin signaling (26). On one hand, moderate activation may contribute to early tissue repair; on the other hand, persistent and excessive activation is a key driver of adverse cardiac remodeling. It promotes fibroblast-to-myofibroblast transition, excessive extracellular matrix deposition, and myocardial fibrosis, resulting in increased ventricular stiffness, impaired contractility, and eventual heart failure (21, 27). As the central downstream effector of this pathway, elevated serum β-catenin may reflect both direct release from damaged cardiomyocytes and robust activation of canonical Wnt signaling within the injured microenvironment. Thus, the increased β-catenin observed in the MACE group likely indicates systemic overactivation of a profibrotic and pro-remodeling signaling network, serving as a “danger signal” of maladaptive repair and poor prognosis.

DKK1, a secreted antagonist that inhibits canonical Wnt activation by binding to LRP5/6 co-receptors (28), may initially rise as a compensatory feedback mechanism. However, growing evidence suggests that persistently high DKK1 levels in chronic inflammatory diseases can induce apoptosis and impair tissue repair. In atherosclerotic plaques, DKK1 overexpression correlates with plaque instability and a higher risk of acute MI (29). Our finding that DKK1 levels were significantly elevated in patients with poor outcomes and positively correlated with MACE risk suggests dysregulated feedback under acute ischemic stress. This observation raises the hypothesis that DKK1 may transition from a compensatory to a potentially detrimental role in the post-STEMI milieu, possibly contributing to pathological processes. However, it is crucial to distinguish this correlative finding from proven causation; our data demonstrate a strong association but do not establish a direct, causal role for DKK1 in MACE pathogenesis. Further mechanistic studies are required to investigate whether elevated DKK1 is merely a marker of severe injury and dysregulated signaling or an active participant in driving adverse outcomes. Conversely, SFRP5, another Wnt antagonist, was markedly reduced in the MACE group, implying diminished inhibition of Wnt signaling and facilitating excessive pathway activation that contributes to adverse outcomes.

Multivariate Cox regression identified β-catenin, DKK1, and cTnI as independent risk factors for MACE. Because single biomarkers often have limited predictive accuracy, we explored the combined prognostic potential of β-catenin (activation marker) and DKK1 (inhibitory marker). This dual-marker strategy achieved superior predictive performance, with an AUC of 0.946—significantly higher than that of individual markers—highlighting their synergistic value in prognostic evaluation. Notably, when β-catenin, DKK1, and cTnI were combined, the AUC increased to 0.969, surpassing both single and dual-marker models. This represents a key novel contribution of our work. This combination suggests that integrating a key pathway activator (β-catenin), its endogenous inhibitor (DKK1), and a terminal injury marker (cTnI) establishes a comprehensive “etiology–compensation–outcome” model, offering a more complete and precise reflection of disease status. This combined model not only outperforms conventional reliance on cTnI alone but also indicates potential for earlier risk detection before irreversible myocardial injury occurs, thereby enabling more accurate risk stratification and personalized intervention.

Nonetheless, several limitations should be acknowledged. First, this was a single-center, retrospective, study with a relatively limited sample size, which may affect generalizability and reduce statistical power for rare outcomes. This design also inherently restricted the availability of certain detailed procedural and anatomical data (e.g., precise time to stent deployment, comprehensive angiographic characteristics) for consistent adjustment in our models. Second, biomarkers were measured at a single time point, preventing assessment of their dynamic changes, which might offer superior prognostic insights. Third, while our modeling strategy mandated adjustment for core demographics and key clinical risk factors, and employed a liberal screening threshold for other confounders, residual confounding from unmeasured or imperfectly captured variables cannot be excluded. Fourth, the precision of hazard ratio estimates for some biomarkers was limited by the number of outcome events, resulting in wider confidence intervals. Fifth, the accuracy of the hazard ratio estimates for individual biomarkers is limited by the number of outcome events, resulting in larger confidence intervals. This highlights the necessity of validation in larger cohorts. Furthermore, the predictive value of our biomarker model was not directly compared with established clinical risk scores such as TIMI or GRACE, which is a necessary step to define its potential incremental utility. Future studies should therefore be prospective in design, enabling the systematic collection of detailed procedural, anatomical, and longitudinal biomarker data. Such multicenter, large-sample cohorts are needed to validate these findings, establish precise cutoff values, and critically evaluate the added predictive value of these biomarkers over existing clinical risk assessment tools.

In conclusion, our findings identify β-catenin and DKK1 within the Wnt/β-catenin pathway as independent risk factors for MACE in STEMI patients. Aberrant activation of this pathway may represent a key pathological mechanism underlying poor prognosis. The combined predictive model based on β-catenin, DKK1, and cTnI significantly improves diagnostic accuracy for MACE, offering a powerful complement to existing assessment systems. More importantly, the novel combined predictive model based on β-catenin, DKK1, and cTnI significantly improves diagnostic accuracy for MACE, offering a powerful complement to existing assessment systems. This model holds promise for earlier risk warning and individualized treatment, providing new biological targets and a theoretical foundation for future precision therapeutic strategies targeting the Wnt/β-catenin signaling pathway.

## Data Availability

The original contributions presented in the study are included in the article/Supplementary Material, further inquiries can be directed to the corresponding author.
